# Co‐viewing as a tool to enhance emotional communication in dementia caregiver–care recipient dyads

**DOI:** 10.1002/alz70858_103117

**Published:** 2025-12-25

**Authors:** Barbara Delacourt, Raed El‐Aoun, Omar Graja, Ahmed Rebei, Santiago Hidalgo, Ana‐Inès Ansaldo

**Affiliations:** ^1^ IUGM research center, Montréal, QC, Canada; ^2^ University of Montreal, Montreal, QC, Canada

## Abstract

**Background:**

Communication difficulties are among the earliest symptoms faced by people living with dementia (PWD), progressively worsening as the disease advances. These challenges often result in reduced interactions with caregivers and a growing sense of isolation for both members of the dyad. Despite awareness of the importance of emotional connection in dementia care, ecological interventions remain limited in long‐term care settings. Studies suggest that co‐viewing audiovisual content with positive emotional valence fosters emotional communication between PWD and caregivers, reduces caregiver burden, and enhances empathy. Our previous studies have also shown that co‐viewing induces physiological synchrony, such as alignment in heart rates and electrodermal activity, between participants (Ansaldo et al., 2024).

**Method:**

This study involved eight PWD and their caregivers, including four family members and four professionals, from a long‐term care facility. Over three weeks, caregivers co‐viewed 20‐minute audiovisual clips with PWD 2–3 times per week. The clips featured themes such as nature, music, and humor, known to evoke positive emotional responses. Semi‐structured interviews were conducted with caregivers to explore the perceived impact of co‐viewing on interactions and emotional connection within the dyad.

**Result:**

Across all caregivers (*N* = 8), co‐viewing significantly enhanced communication and socialization within the dyads. The activity elicited verbal and nonverbal reactions from PWD, fostering meaningful exchanges. Professional caregivers reported that the sessions helped them better understand and connect with the residents, while family caregivers found the intervention provided a much‐needed communication tool to navigate challenging interactions with their loved ones. Caregivers observed positive effects on both themselves and PWD, noting feelings of enjoyment and calmness during the sessions. They highlighted that PWD were actively engaged in the activity, as evidenced by their reactions and sustained attention to the screen, which contrasted with their usual state of apathy.

**Conclusion:**

These findings underscore the potential of co‐viewing as a strategy to promote emotional communication within caregiver–PWD dyads. Its simplicity and adaptability make it a promising intervention for broader implementation in long‐term care settings. Future studies will analyze specific communication behaviors within dyads during co‐viewing sessions.

